# An Adaptive Real-Time Detection Algorithm for Dim and Small Photoelectric GSO Debris

**DOI:** 10.3390/s19184026

**Published:** 2019-09-18

**Authors:** Quan Sun, Zhaodong Niu, Weihua Wang, Haijing Li, Lang Luo, Xiaotian Lin

**Affiliations:** 1 National Key Laboratory of Science and Technology on ATR, National University of Defense Technology, Changsha 410073, China; 2 China Xi’an Satellite Control Center, Xi’an 710000, China

**Keywords:** dim and small GSO debris, CCD image, adaptive fast registration, enhanced dilation difference, mathematical morphology, multi-target tracking

## Abstract

Geosynchronous orbit (GSO) is the ideal orbit for communication, navigation, meteorology and other satellites, but the space of GSO is limited, and there are still a large number of space debris threatening the safety of spacecraft. Therefore, real-time detection of GSO debris is necessary to avoid collision accidents. Because radar is limited by transmitting power and operating distance, it is difficult to detect GSO debris, so photoelectric detection becomes the mainstream way to detect GSO debris. This paper presents an adaptive real-time detection algorithm for GSO debris in the charge coupled device (CCD) images. The main work is as follows: An image adaptive fast registration algorithm and an enhanced dilation difference algorithm are proposed. Combining with mathematical morphology, threshold segmentation and global nearest neighbor (GNN) multi-target tracking algorithm, the functions of image background suppression, registration, suspected target extraction and multi-target tracking are realized. The processing results of a large number of measured data show that the algorithm can detect dim geostationary earth orbit (GEO) and non-GEO debris in GSO belt stably and efficiently, and the processing speed meets the real-time requirements, with strong adaptive ability, and has high practical application value.

## 1. Introduction

The GSO belt is 42,164 kilometers away from the center of the earth, 400 kilometers in radial thickness and delimited by ±15° in latitude [1]. It is an ideal orbit for communications, navigation, meteorology and other satellites [2,3]. According to data released by the U.S. Strategic Command, as of July 2018, there were about 1200 objects in GSO orbit and about 400 satellites in service [1,4]. In addition, according to data from the European Space Agency, as of January 2019, there were about 1950 spacecraft in normal working state in all Earth orbits. At the same time, it was estimated that about 129 million debris were larger than 1mm, while only about 22.3 thousand objects were catalogued in the database and could be continuously tracked. Although the density of debris in GSO is much less than that in low Earth orbit (LEO), there is no debris clearance mechanism similar to atmospheric damping in GSO; debris will persist for millions of years and pose a greater threat to spacecraft [5,6]. Maintaining space security and accurately monitoring space debris is a part of space situational awareness [7]. Accurate monitoring of GSO objects can judge the operational status of satellites, evaluate the probability of collision events, and contribute to celestial mechanics, help people understand orbit evolution and motion characteristics [8,9]. Therefore, it is meaningful to study the detection algorithm of GSO debris [10,11]. The detection methods of space objects mainly include radar detection and optical detection. Microwave radar has the advantages of high accuracy and all-weather, but its maximum operating distance is proportional to the fourth power of transmitting power. Therefore, it is difficult to detect GSO debris with radar, which requires huge transmitting power and large antenna size [12,13]. In view of this, optical detection has become the mainstream method of GSO debris detection. Optical detection of GSO debris has been carried out for decades [14]. With the improvement of optical sensor performance, the sensitivity of optical detection is getting higher and higher. Cohen et al. successfully observed GSO objects in daytime with two different biologically-inspired cameras [15]. In addition, a 2-D GSO objects image with a resolution of 0.1 m can be obtained by combining inverse synthetic aperture lidar (ISAL) [16].

Generally, when the telescope performs the task of optical detection of space objects, there are two kinds of working modes, stellar tracking and target tracking. Stellar tracking mode is usually used to detect LEO objects. GSO objects detection generally uses target tracking mode. Because, relative to ground-based telescopes, GSO debris mainly includes GEO debris which is approximately stationary, and non-GEO debris with uniform low-speed linear motion, the target tracking mode here actually refers to fixing the telescope field of view in the target area [17]. In CCD images, target tracking mode results in stripe-like images of stars, whereas GEO debris are dot images, and non-GEO debris are stripe-like images with different lengths or directions of stellar stripes. Ultra-long-distance observation results in the weak intensity and small size of GSO debris in CCD images. Therefore, it is a typical “dim”, “small” and “slow” target. It is a challenge to detect GSO debris accurately. In addition, because of the gravitational field disturbance of the sun, the moon and the earth, as well as the solar radiation pressure disturbance, the intensity of GSO debris in the image and the orbit are time-varying. The change of intensity leads to discontinuity of target extraction results, which is a challenge to both target extraction and track association algorithms. Orbital variations make it difficult to predict their trajectories for more than a week, requiring enhanced GSO debris continuous monitoring capacity [18,19,20,21,22].

GSO debris detection in CCD image mainly involves targets extraction and track association. GSO debris extraction algorithms can be divided into feature extraction based on shape difference and target enhancement through multi-frame cumulative energy. The main methods are masking method [23], continuous frame image comparison method [24], point spread function fitting method [25], mathematical morphology method [26] and so on. Generally, feature extraction algorithms have high complexity and require prior feature information of target or background; multi-frame energy accumulation algorithm needs to ensure efficient suppression of stars and background. Considering the real-time performance and adaptability of the algorithm, this paper proposes an adaptive fast registration algorithm and an enhanced dilation difference algorithm for GSO debris extraction. The track association of GSO debris belongs to the field of multi-target tracking. For multi-target tracking, there are two main types of algorithms based on random finite set (RFS) and data association, respectively. Multi-target tracker based on RFS includes unlabeled RFS type and labeled RFS type. Unlabeled RFS type lacks target discrimination, while labeled RFS type increases the complexity of the algorithm. The biggest problem is that in the application of GSO debris optical detection, the birth model of targets is difficult to be given, while the typical RFS tracker cannot work without the birth model [27,28,29,30]. Multi-target trackers based on data association include GNN [31] (pp. 203–205), joint probabilistic data association (JPDA) [32], multiple hypothesis tracking (MHT) [33] tracker and their variants. JPDA and MHT trackers are superior to GNN tracker in complex tracking scenarios, but their computational complexity will increase dramatically. The motion form of GSO debris is relatively simple: approximately stationary or uniformly low-speed linear motion, so GNN tracker can complete the tracking task well. Considering comprehensively, we choose GNN tracker to track GSO debris. The measured data processing results also verify that the GNN tracker can accomplish the tracking task well, and the computational complexity is low.

In this paper, an adaptive real-time detection algorithm for dim and small photoelectric GSO debris is proposed. The algorithm has the advantages of low computational complexity, excellent detection performance and strong adaptive ability. The algorithm consists of targets extractor and tracker. Target extractor has the following parts: background suppression based on the morphological Top-Hat transform algorithm, image registration based on the adaptive fast registration algorithm, stellar suppression based on the enhanced dilation difference algorithm, GSO debris enhancement and target segmentation based on inter-frame correlation and threshold segmentation technology. The target tracker uses GNN multi-target tracker and can estimate the inter-frame interval adaptively to improve the performance of the tracker.

The rest of the paper is organized as follows. In the second section, each component of GSO debris extractor is introduced, and the adaptive fast registration algorithm and the enhanced dilation difference algorithm proposed in this paper are emphatically introduced. In the third section, the GSO debris tracker is introduced. The principle of GNN tracker and the adaptive inter-frame interval estimation technique proposed in this paper are described. The fourth section contains the introduction of test data and the processing results of the algorithm. The fifth section gives the conclusions and the prospects of future work.

## 2. GSO Debris Extractor

This section will introduce each component of GSO debris extractor, including background suppression, image registration, stellar suppression, GSO debris enhancement and target segmentation.

### 2.1. Background Suppression Based on Morphological Top-Hat Transform

The background of CCD star image will be disturbed by various noises, including photon noise, environmental noise, readout noise and so on. These interferences seriously affect the effect of subsequent image processing steps, so they must be suppressed. In view of the strong image adaptive ability of mathematical morphology, parallel processing and fast computing speed [34], this paper uses morphological Top-Hat transform algorithm for background suppression. Top-Hat transform background suppression algorithm has been widely used because of its advantages in background suppression and real-time operation. The formula of top-hat transform is as follows:

(1) g=f−(f∘b),

where f is the original input image and b is the structural element.f∘b stands for open operation of image f using structural element b, which is equivalent to a non-linear low-pass filter. It can remove bright details smaller than the size of structural element, while preserving the overall gray value of the image, and the larger bright areas are almost unaffected. The background of the image can be estimated by using appropriate structure element b to perform open operation. The g obtained by subtraction is the target image that suppresses the background.

**Figure 1:**
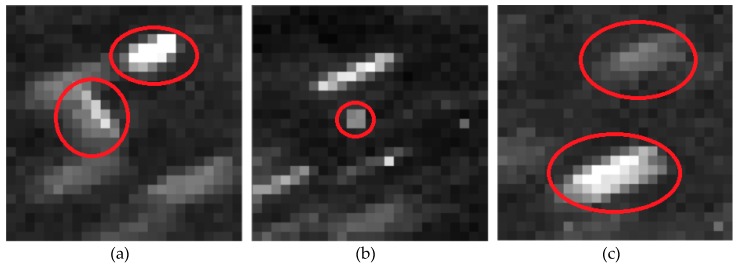
Composition of charge coupled device (CCD) star image besides background: (**a**) non- geostationary earth orbit (GEO) debris; (**b**) GEO debris; (**c**) stars.

Whether the selection of structural element is appropriate or not will have a great impact on the processing efficiency and effect of Top-Hat transformation. According to the principle, the size of structural element should be larger than the targets size. By analyzing the measured data, the CCD star image mainly includes GEO debris, non-GEO debris and stars besides background noise, as shown in Figure 1.

**Figure 2:**
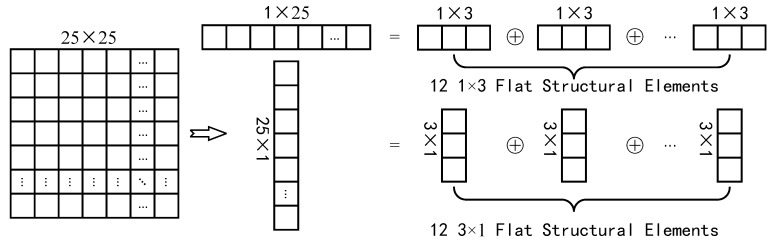
Schematic diagram of structural element decomposition.

**Figure 3:**
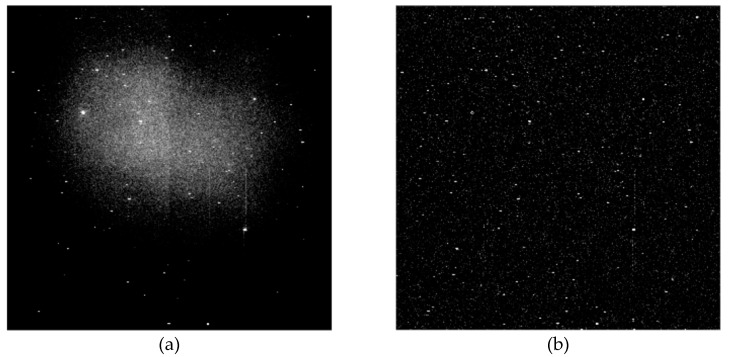
CCD star image before and after background suppression: (**a**) before background suppression; (**b**) after background suppression.

In the image data used in this paper, the size of GSO debris is between 2×2 and 20×20 pixels. In order to reduce the computational complexity as much as possible while guaranteeing better background suppression effect, the size of the structural element used in this algorithm is 25×25, and it is further decomposed into flat structural elements of 1×25 and 25×1 [35]. The erosion and dilation operations of flat structural elements involved in the open operation are simplified to the minimum and maximum filters with the support domain of structural elements as the window respectively, which greatly reduces the algebraic computation. In order to further improve the processing speed and take into account the portability of the algorithm, flat structure elements of 1×25 and 25×1 are decomposed into small size structure elements of 1×3 and 3×1 [36], as shown Figure 2. If the image size is m×n, the computational complexity of the background suppression algorithm is O(mn). Figure 3 shows the CCD star images before and after background suppression. It can be seen that the background is effectively suppressed.

### 2.2. Adaptive Fast Image Registration

**Figure 4:**
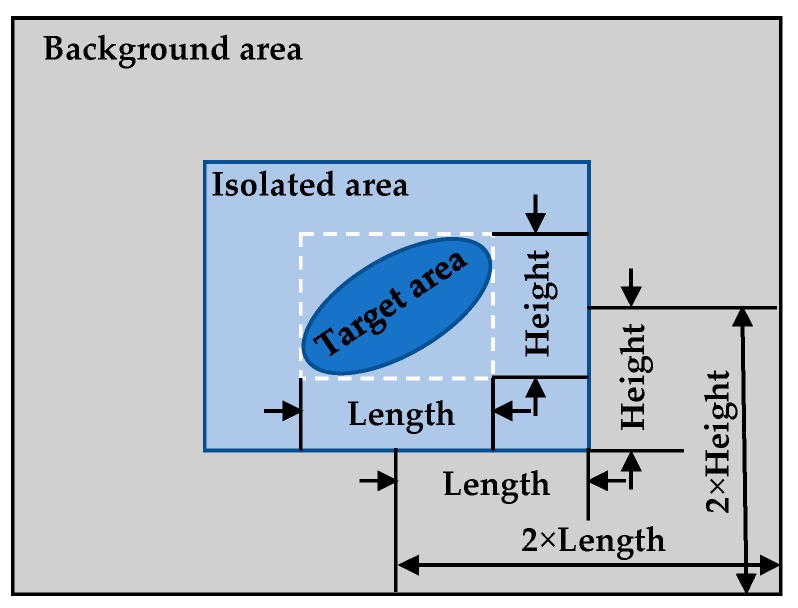
Definition of correlation region of signal-to-noise ratio (SNR).

Because GSO debris are typical dim and small targets, in order to improve the probability of detection, an effective way is to accumulate energy through multi-frame images to improve the signal-to-noise ratio (SNR) of the target. In this paper, local SNR is adopted, which is defined as follows:

(2) $$SNR=\left(f_{T}-f_{B}\right)/\sigma ,$$

where, $f_{T}$ and $f_{B}$ are the gray mean of the target area and background area respectively, and σ is the gray mean variance of the background area. In order to prevent the interference between target and background gray statistics, they are separated by isolation area. The definitions of different regions are shown in Figure 4.

The premise of multi-frame image processing is image registration. GSO debris detection uses ground-based telescope, and the tracking mode of the telescope is the target tracking mode. Because the GSO debris moves slowly relative to the ground-based telescope, the target tracking mode here actually means that the telescope field of view is fixed in the target sky area, that is, the telescope is stationary when it is working normally. Therefore, the movement between the two frames is basically caused by the motion of the stars. The motion of stars is mainly caused by the rotation of the earth, the rotation speed of the earth is basically constant, and because the stars are very far away from the telescope. So, on the CCD image, there is only the translational motion of the stars, and there is no rotation. Overall, for the registration of CCD star images of GSO only involves translation transformation, and often lacks information such as exposure time, telescope position, and optical axis pointing. In view of this, an adaptive fast registration algorithm is proposed in this paper. The correlation coefficient is a statistical index to reflect the degree of close correlation between variables. Obviously, when the correlation coefficient of two images is the largest, it can be judged that two images have been registered. Therefore, our goal is to find a translation vector $u_{shift}=\left(x_{shift},y_{shift}\right)$ to maximize the correlation coefficient between the shifted image $I_{shift}$ and the reference image $I_{fixed}$. The formula for calculating the correlation coefficient is as follows:

(3) $$\rho \left(u_{shift}\right)=\frac{\sum \sum \left(I_{shift}\left(u_{shift}\right)-E\left(I_{shift}\left(u_{shift}\right)\right)\right)\left(I_{fixed}-E\left(I_{fixed}\right)\right)}{\sqrt{\left(\sum \sum {\left(I_{shift}\left(u_{shift}\right)-E\left(I_{shift}\left(u_{shift}\right)\right)\right)}^{2}\right)\left(\sum \sum {\left(I_{fixed}-E\left(I_{fixed}\right)\right)}^{2}\right)}},$$

where $I_{shift}\left(u_{shift}\right)$ represents the shifted image after translation according to translation vector $u_{shift}$, $\rho \left(u_{shift}\right)$ represents the corresponding correlation coefficient, E(⋅) represents the average value of the image.

**Figure 5:**
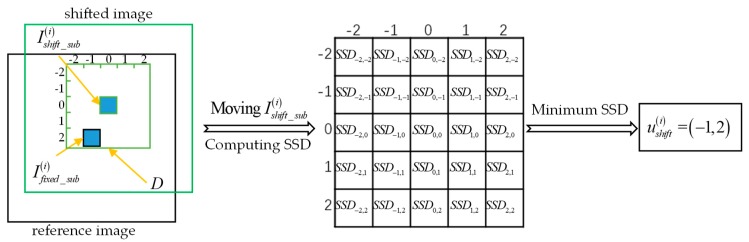
The procedure of calculating the translational vector of the i-th sub-image.

The maximum correlation coefficient can be measured by minimizing the sum of squared differences (SSD) of two images [37]. The formula for calculating the SSD is as follows:

(4) $$SSD\left(u_{shift}\right)=\sum \sum {\left(I_{shift}\left(u_{shift}\right)-I_{fixed}\right)}^{2},$$

where $SSD\left(u_{shift}\right)$ represents the SSD corresponding to translation vector $u_{shift}$. When the size of the image is large, the amount of calculation to get the SSD of the whole image is very large, which seriously reduces the processing speed. In order to improve the efficiency, we uniformly select n identical sub-images on the reference image and the shifted image, in which the sub-images on the reference image is fixed and unchanged, and the sub-images on the shifted image moves within a given search area, each moving step is one pixel. At each step, the SSD of the corresponding sub-images is calculated. After all SSDs corresponding to the search area are calculated, the translation vector corresponding to the minimum SSD is required. Let D denote the search area, $I_{fixed\_sub}^{\left(i\right)}$ and $I_{shift\_sub}^{\left(i\right)}$ denote the i-th sub-image selected from the reference image and the shifted image respectively, and $u_{shift}^{\left(i\right)}$ denote the translation vector determined by the i-th sub-image, then the calculation formula of $u_{shift}^{\left(i\right)}$ is as follows:

(5) $$u_{shift}^{\left(i\right)}=Find\left(\min\left(\left\{\sum \sum {\left(I_{shift\_sub}^{\left(i\right)}\left(u_{shift}\right)-I_{fixed\_sub}^{\left(i\right)}\right)}^{2};u_{shift}\in D\right\}\right)\right),$$

where function Find(⋅) obtains the translation vector corresponding to SSD. Figure 5 shows how to calculate the translation vector of the i-th sub-image. For clarity, the size of D is assumed to be 5×5 and the translation vector $u_{shift}^{\left(i\right)}=\left(-1,2\right)$ in Figure 5. Obviously, using this method, the computational complexity is only related to the number and size of selected sub-images, and the size of search area, which is independent of the size of the whole image, and the algorithm has good parallel processing characteristics. When the number of sub-images is $n_{s}$, the size of each sub-image is $m_{s}\times m_{s}$, and the size of search area is $r_{s}\times r_{s}$, then, the computational complexity is $O\left(n_{s}m_{s}^{2}r_{s}^{2}\right)$.

An intuitive way to obtain the translation vector of the whole image is to directly calculate the mean of the translation vector determined in all sub-images. This method is feasible in most cases, but because the size of the selected sub-image is much smaller than that of the whole image, the translation vector determined in the sub-image may have a larger error with the actual translation vector of the whole image. The results of the measured data processing also show that there are such problems. In order to overcome this problem, we noticed that the translation vector determined in most sub-images are basically the same as the translation vector of the whole image. Therefore, we classify all translation vectors determined in sub-images according to the 2-norm (Euclidean distance) of the difference between translation vectors, and calculate the translation vector of the whole image by using the class with the largest number of translation vectors. The implementation process is as follows:

The first step is to calculate the Euclidean distance $d_{shift}^{\left(i,j\right)}$ between the translation vector $u_{shift}^{\left(i\right)}$ determined in the i-th sub-image and the translation vector $u_{shift}^{\left(j\right)}\left(j\neq i\right)$ determined in other sub-images.

(6) $$d_{shift}^{\left(i,j\right)}={\left\|u_{shift}^{\left(i\right)}-u_{shift}^{\left(j\right)}\right\|}_{2},\;i\neq j,$$

where ${\left\|\cdot \right\|}_{2}$ denotes 2-norm.

In the second step, all translation vectors whose distances from $u_{shift}^{\left(i\right)}$ are less than threshold $T_{shift}$ are classified into categories determined by $u_{shift}^{\left(i\right)}$ and expressed in $U_{shift}^{\left(i\right)}$, and the number of translation vectors contained in $U_{shift}^{\left(i\right)}$ is calculated as follows:

(7) $$U_{shift}^{\left(i\right)}=\left\{d_{shift}^{\left(i,j\right)}|d_{shift}^{\left(i,j\right)}\leq T_{shift},j=1,\cdots ,n\;\mathrm{and}\;i\neq j\right\},$$

(8) $$N_{shift}^{\left(i\right)}=\left|U_{shift}^{\left(i\right)}\right|,$$

where $N_{shift}^{\left(i\right)}$ represents the number of elements contained in $U_{shift}^{\left(i\right)}$ and |⋅| represents the potential (the number of elements) of the set. Because the search step is one pixel, the accuracy of the translation vectors is one pixel in both horizontal and vertical directions, so the distance threshold $T_{shift}$ is set to $\sqrt{2}$.

The third step is to perform the same operation on all translation vectors determined in sub-images.

In the fourth step, the translation vector of the whole image is calculated according to the $U_{shift}^{\left(M\right)}$ corresponding to the maximum $N_{shift}^{\left(M\right)}$.

(9) $$N_{shift}^{\left(M\right)}=\max\left(\left\{N_{shift}^{\left(i\right)};i=1,\cdots ,n\right\}\right);U_{shift}^{\left(M\right)}=Find\left(N_{shift}^{\left(M\right)}\right),$$

(10) $$u_{shift}=\sum \left\{U_{shift}^{\left(M\right)},u_{shift}^{\left(i\right)}\right\}/\left(N_{shift}^{\left(M\right)}+1\right),$$

By using the above method, reliable translation vectors of the whole image can be obtained. However, considering the sudden change of exposure time in order to adapt to the change of targets’ intensity when using telescopes to observe GSO debris, it is difficult to determine the search area. It is often necessary to give a larger area to satisfy different exposure times as much as possible, which leads to a significant increase in processing time. In addition, it should be noted that the exposure time will not change frequently on a large scale. Due to the instability of device state and other factors, it usually changes in a very small range, so we can reduce the search area to reduce processing time.

In order to take into account the above two situations, an adaptive search strategy is proposed in this paper. Initially, the translation vector $u_{o}$ is determined by using large search area $D_{\mathrm{large}}$. According to the exposure time, we set $D_{\mathrm{large}}$ as a 100×100 rectangular area centered on the origin. The processing steps of subsequent frames are as follows:

In the first step, the translation vector $u_{k-1}$ determined in the previous frame is directly used to extract suspected targets. It can be considered that the search area in this step is $D_{1}$ with size 1×1, and the extraction algorithm will be introduced in Section 2.3 and Section 2.4. If the number of suspected targets is less than the threshold $T_{SusObj}$, the translation vector $u_{k-1}$ is judged to be correct and the registration is completed. Also, make $u_{k}=u_{k-1}$. Otherwise, the translation vector is judged to be wrong.

In the second step, if $u_{k-1}$ is wrong, considering that the accuracy of the translation vector is one pixel in both horizontal and vertical directions, and the exposure time of the telescope often varies in a small range, then the search area is enlarged to 5×5 area centered on $u_{k-1}$ and marked as $D_{2}$. The translation vector ${\overset{⌢}{u}}_{2}$ is obtained by searching area $D_{2}$, and the suspected targets are extracted by ${\overset{⌢}{u}}_{2}$. If the number of suspected targets met the quantitative constraint, less than the threshold $T_{SusObj}$ and the translation vector satisfied the translation constraint $R_{u}$, then the translation vector ${\overset{⌢}{u}}_{2}$ is judged to be correct and the registration is completed. Also, make $u_{k}={\overset{⌢}{u}}_{2}$. Otherwise, the translation vector is judged to be wrong. How to determine the threshold $T_{SusObj}$ and translation vector constraint $R_{u}$ will be introduced later.

In the third step, if ${\overset{⌢}{u}}_{2}$ is wrong, considering that the exposure time may vary in a wide range in order to adapt to the change of target intensity, the change range is generally half of the original exposure time to twice the original exposure time. The search area is enlarged to $2\left|u_{k-1}\_x\right|\times 2\left|u_{k-1}\_y\right|$ area centered on $u_{k-1}$ and recorded as $D_{3}$, where, $u_{k-1}\_x$ and $u_{k-1}\_y$ represent x and y coordinates of $u_{k-1}$ respectively. The translation vector ${\overset{⌢}{u}}_{3}$ is obtained by searching area $D_{3}$, and the suspected targets are extracted by ${\overset{⌢}{u}}_{3}$. Similarly, if the number of suspected targets is less than the threshold $T_{SusObj}$ and the translation vector satisfied the translation constraint $R_{u}$, the translation vector ${\overset{⌢}{u}}_{3}$ is judged to be correct and the registration is completed. Also, make $u_{k}={\overset{⌢}{u}}_{3}$. Otherwise, the translation vector is judged to be wrong.

**Figure 6:**
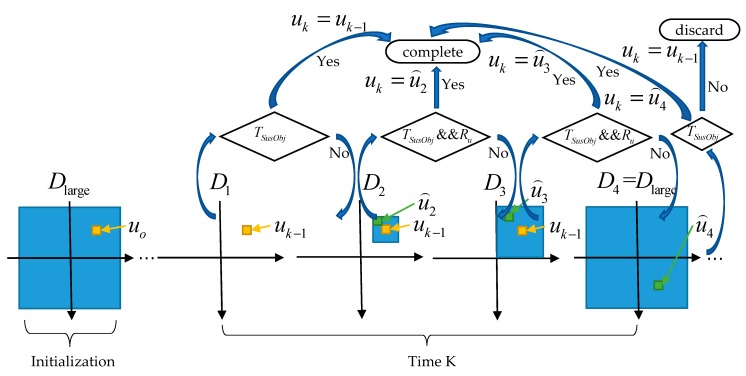
The search strategy schematic.

In the fourth step, if ${\overset{⌢}{u}}_{3}$ is wrong, consider that the abnormal change of exposure time may be caused by abnormal reasons. The translation vector ${\overset{⌢}{u}}_{4}$ is obtained by adjusting the search area to $D_{4}=D_{\mathrm{large}}$, and then suspected targets are extracted by ${\overset{⌢}{u}}_{4}$. The threshold $T_{SusObj}$ is used to judge whether ${\overset{⌢}{u}}_{4}$ is correct or not. Note that the step does not need translation constraint $R_{u}$, because the exposure time is likely to be abnormal at this time, so it is likely that the correct ${\overset{⌢}{u}}_{4}$ does not meet the translation constraint. If ${\overset{⌢}{u}}_{4}$ is correct, registration is completed and $u_{k}={\overset{⌢}{u}}_{4}$. If ${\overset{⌢}{u}}_{4}$ is wrong, consider that it may be due to the change of the telescope field of view, or a large amount of thick clouds occlusion that cannot be registered, then stop searching and discard the frame. Also, make $u_{k}=u_{k-1}$. The search strategy schematic is shown in Figure 6.

Through the analysis of a large number of measured data, we found that the number of GSO debris in CCD star image generally does not exceed 15, and the number of false targets extracted from a single frame image by the target extractor in this paper generally does not exceed 10. Therefore, the threshold $T_{SusObj}$ of quantitative constraints is set to 40. For translation constraint $R_{u}$, it should be noted that the processing of each frame image involves two registration processes, including the registration of the previous frame image and the current frame image, whose registration translation vector is marked as $u_{shift\_1}$, the registration of the first two frames with the current frame image, and the registration displacement vector is marked as $u_{shift\_2}$. Considering that the stellar translation is generally greater than 5 pixels and less than 50 pixels during the exposure period of GSO debris optical observation, the first constraint of $R_{u}$ is $5\leq {\left\|u_{shift\_1}\right\|}_{2}\leq 50$ and $10\leq {\left\|u_{shift\_2}\right\|}_{2}\leq 100$. In addition, in order to improve robustness, we add the ratio of two translation vector modulus as the second constraint condition. Normally, the interval of changing exposure time to adapt to the change of target intensity is usually 0.5 to 2 times of the original exposure time. Considering that both horizontal and vertical accuracy of translation vectors are 1 pixel, the maximum value M_R of the ratio of modulus of two translation vectors can be determined by the following formula.

(11) $$\left\{ \begin{array}{r} M\_R=\sqrt{\max\left(k^{2}\left({\left(x+1\right)}^{2}+{\left(y+1\right)}^{2}\right)/\left(x^{2}+y^{2}\right)\right)}\\ 0\leq x\leq 50,0\leq y\leq 50,x^{2}+y^{2}\geq 25,1.5\leq k\leq 3 \end{array}\right.,$$

and determine the minimum value m_R of the ratio of modulus of two translation vectors by the following formula:

(12) $$\left\{ \begin{array}{r} m\_R=\sqrt{\min\left(k^{2}\left({\left(x-1\right)}^{2}+{\left(y-1\right)}^{2}\right)/\left(x^{2}+y^{2}\right)\right)}\\ 0\leq x\leq 50,0\leq y\leq 50,x^{2}+y^{2}\geq 25,1.5\leq k\leq 3 \end{array}\right.,$$

Using the software of MATLAB, we get M_R≈14.811, m_R≈2.3409. The above image registration algorithm, combined with the specific application of GSO debris optical detection, takes into account most of the situations in image registration, has the characteristics of strong adaptive ability, fast computing speed and parallel processing.

### 2.3. Stellar Suppression Based on the Enhanced Dilation Difference Algorithm

**Figure 7:**
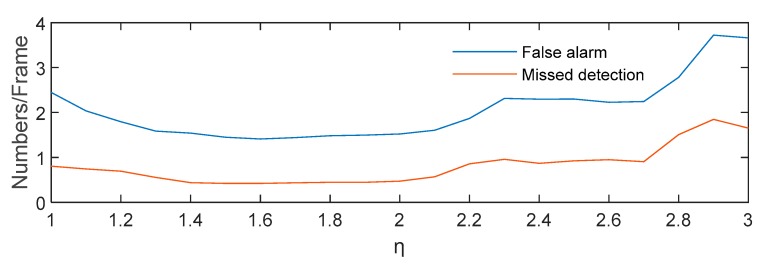
The change of average false alarm and missed detection number per frame with η.

**Figure 8:**
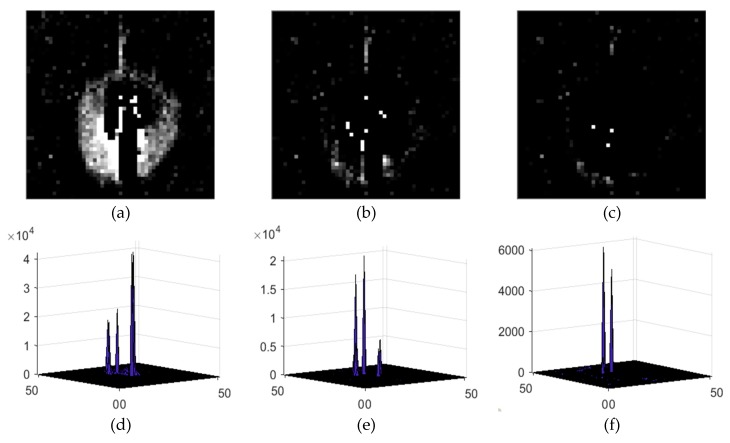
Result image and corresponding gray distribution of different differential algorithms: (**a**) absolute difference result image; (**b**) dilation difference result image; (**c**) enhanced dilation difference result image (η=2); (**d**) absolute difference gray distribution; (**e**) dilation difference gray distribution; (**f**) enhanced dilation difference gray distribution.

The stellar suppression algorithm based on enhanced dilation difference is an improved algorithm proposed in this paper to overcome the shortcomings of dilation difference algorithm. GSO debris detection generally adopts target tracking mode, so the translation of all stars in CCD images is basically the same, and the motion mode of GSO debris is different from that of stars whether GEO or non-GEO objects, so we can suppress stars by using inter-frame difference technology after image registration. There are two main problems to be solved in the suppression of stars by inter-frame difference technology. Firstly, using adaptive fast image registration algorithm, the accuracy of translation vector in horizontal and vertical directions is 1 pixel. Therefore, if absolute difference is used directly, there will be residual marks on the edge, even if the translation vector obtained is accurate, because there are many stars, there are small differences in their translation, and the difference results will also leave edge remnants. In particular, when GSO observations are performed, the camera usually does not have a mechanical shutter, which results in a serious smear of high-brightness stars, and the edge remnants left by the smear will seriously affect the subsequent target segmentation effect. Secondly, the intensity of stars varies in time because of the interference of various internal and external factors, that is, the intensity of the same star in the first and second frames is generally inconsistent, especially the intensity of high-brightness stars is often very different, which leads to the difference results leaving the remnants of stars. The remnants of high-brightness stars can be severe and can even lead to the inability to segment targets. For the first problem, we can use the dilation difference algorithm to solve [38]. Dilation difference first dilates the subtracted image using structural element b. Considering the accuracy of the registration algorithm, this paper chooses b as a flat structure element of 3×3. Let $I_{0}\left(x,y\right)$ be the intensity of the image at (x,y) after background suppression of the current frame, and $I_{1}\left(x,y\right)$ be the intensity of the image at (x,y) after background suppression of the previous frame and already registered with the current frame. The dilation difference formula is as follows:

(13) $$I_{d\_01}\left(x,y\right)=\left\{ \begin{array}{r} I_{0}\left(x,y\right)-\left(I_{1}\left(x,y\right)⊕b\right)\begin{array}{cc} , & if \end{array}\begin{array}{cc} I_{0}\left(x,y\right)>I_{1}\left(x,y\right) & \end{array}\\ \begin{array}{cccc} & & & \begin{array}{cc} \begin{array}{cc} 0 & \end{array} & \begin{array}{cc} , & if \end{array}\begin{array}{cc} I_{0}\left(x,y\right)\leq I_{1}\left(x,y\right) & \end{array} \end{array} \end{array} \end{array}\right.,$$

where $I_{d\_01}\left(x,y\right)$ represents the intensity of the image at (x,y) of the dilation difference result, “⊕” is the dilation operator. Dilation difference algorithm solves the problem of stellar edge remnants caused by scale mismatch. For the second problem, we need to solve the problem of stellar remnants caused by the difference of stellar intensity. The specific reason is that the intensity of the star in the current frame is stronger than that in the previous frame. In order to solve this problem, we propose multiplying an intensity enhancement factor η after the dilation operation of the previous image. It should be pointed out that the same GSO debris will not be in the same position in the previous frame and the current frame image after registration because of the difference between the motion mode of GSO debris and the stars, while the enhancement factor only changes the gray value of the image, so the enhanced dilation difference operation will not suppress the GSO debris. The enhanced dilation difference formula is as follows:

(14) $$I_{ed\_01}\left(x,y\right)=\left\{ \begin{array}{r} I_{0}\left(x,y\right)-\eta \left(I_{1}\left(x,y\right)⊕b\right)\begin{array}{cc} , & if \end{array}\begin{array}{cc} I_{0}\left(x,y\right)>I_{1}\left(x,y\right) & \end{array}\\ \begin{array}{cccc} & & 0 & \begin{array}{cc} & \begin{array}{cc} , & if \end{array}\begin{array}{cc} I_{0}\left(x,y\right)\leq I_{1}\left(x,y\right) & \end{array} \end{array} \end{array} \end{array}\right.,$$

where $I_{ed\_01}\left(x,y\right)$ represents the intensity of the image at (x,y) after the enhanced dilation difference operation, and the enhancement factor η generally ranges from 1.5 to 2. In order to determine the range of η, the average number of false alarms and missed detections per frame of 496 images in seven image sequences is counted when η=1:0.1:3, as shown in Figure 7. Thus, when 1.5≤η≤2, the number of false alarms and missed detection is relatively small. In addition, according to [39,40,41], the magnitude of a variable star will not change more than 0.75 in 5 to 15 seconds of exposure time, that is, the brightness of the same variable star is less than twice that of the previous frame. In order to show the effect of the algorithm, the result image and the corresponding gray distribution of the same high-brightness star after the operation of absolute difference, dilation difference and enhanced dilation difference are given, as shown in Figure 8.

### 2.4. GSO Debris Enhancement and Target Segmentation Based on Inter-Frame Correlation and Threshold Segmentation Technology

This section contains two processing steps, GSO debris enhancement and target segmentation, but because the two operations are closely related, we did not discuss them separately for ease of understanding. In view of the weak SNR of GSO debris on CCD image, there are two main methods to improve the SNR of the target. The first method is to prolong the exposure time. This strategy is indeed used in the GSO debris observation task. However, it should be noted that exposure time is limited. When the exposure time is too long, it will cause the saturation overflow of CCD sensor pixels. In addition, the long exposure time only improves the SNR of the GEO debris, but has little effect on the non-GEO debris. The second method is to accumulate energy by using multiple frames. This paper uses the inter-frame correlation processing algorithm to achieve this goal. Firstly, the current frame and the previous frame, and the current frame and the first two frames are used to obtain two differential images $I_{ed\_01}$ and $I_{ed\_02}$ respectively by using the enhanced dilation difference algorithm.

(15) $$I_{ed\_01}\left(x,y\right)=\left\{ \begin{array}{r} I_{0}\left(x,y\right)-\eta \left(I_{1}\left(x,y\right)⊕b\right)\begin{array}{cc} , & if \end{array}\begin{array}{cc} I_{0}\left(x,y\right)>I_{1}\left(x,y\right) & \end{array}\\ \begin{array}{cccc} & & 0 & \begin{array}{cc} & \begin{array}{cc} , & if \end{array}\begin{array}{cc} I_{0}\left(x,y\right)\leq I_{1}\left(x,y\right) & \end{array} \end{array} \end{array} \end{array}\right.,$$

(16) $$I_{ed\_02}\left(x,y\right)=\left\{ \begin{array}{r} I_{0}\left(x,y\right)-\eta \left(I_{2}\left(x,y\right)⊕b\right)\begin{array}{cc} , & if \end{array}\begin{array}{cc} I_{0}\left(x,y\right)>I_{2}\left(x,y\right) & \end{array}\\ \begin{array}{cccc} & & 0 & \begin{array}{cc} & \begin{array}{cc} , & if \end{array}\begin{array}{cc} I_{0}\left(x,y\right)\leq I_{2}\left(x,y\right) & \end{array} \end{array} \end{array} \end{array}\right.,$$

Then, multiplying the two differential images can enhance the GSO debris and further suppress the stars:

(17) $$I_{multip\_0}\left(x,y\right)=I_{ed\_01}\left(x,y\right)*I_{ed\_02}\left(x,y\right),$$

where $I_{multip\_0}$ represents the result of the current frame processed by inter-frame correlation. At this point, we get the background and stars are effectively suppressed, the GSO debris are effectively enhanced image, then we can extract the suspected GSO debris on $I_{multip\_0}$.

In this paper, threshold segmentation technology is used to extract GSO debris. Threshold segmentation is mainly divided into global threshold segmentation and local threshold segmentation. Generally speaking, the performance of segmentation algorithm based on local threshold is better than that of global threshold, but the complexity of the algorithm is much higher than that of global threshold. In view of the fact that the background and stars in the CCD star image have been effectively suppressed based on the preprocessing algorithm mentioned above, in order to improve the processing speed, we choose to segment and extract GSO debris using global thresholds. Firstly, the segmentation threshold is calculated as follows:

(18) $$T_{segmen}=E\left(I_{multip\_0}\right)+\kappa \times SD\left(I_{multip\_0}\right),$$

where, $E\left(I_{multip\_0}\right)$ represents the mean of the image, $SD\left(I_{multip\_0}\right)$ represents the standard deviation of the image, and κ is the adjustment coefficient, which is determined by the minimum SNR of the detectable target. For dim and small GSO debris segmentation, in order to improve detection probability, κ is usually set as small as possible, but at the same time, it also increases the false alarm probability when extracting suspected targets, which challenges how to efficiently complete GSO debris track association. After the segmentation threshold is determined, the image is segmented by the threshold, and the binary image $I_{binary\_0}$ is obtained as follows:

(19) $$I_{binary\_0}=\left\{ \begin{array}{c} \begin{array}{cc} & \begin{array}{cc} 1 & \begin{array}{cc} , & \begin{array}{cc} if & I_{multip\_0}\geq T_{segmen} \end{array} \end{array} \end{array} \end{array}\\ \begin{array}{cc} & \begin{array}{cc} 0 & \begin{array}{cc} , & \begin{array}{cc} if & I_{multip\_0}<T_{segmen} \end{array} \end{array} \end{array} \end{array} \end{array}\right.,$$

For the binary image $I_{binary\_0}$, the connected area is searched according to the 8-connected objects, and each connected component is labeled. Finally, the centroid of each suspected target area is calculated. Generally, there are three kinds of suspected targets: GEO debris, non-GEO debris and false targets. The image of an GEO debris is a circular dim region. The image of a non-GEO debris appears as a dim strip or ellipse. False targets are usually incompletely suppressed stars, and their images are long strips, but their lengths or directions are generally different from those of non-GEO debris. There are two methods for calculating the centroid of an object image: fitting method and modified moment method. Fitting method has high positioning accuracy, but its calculation is huge, and its accuracy depends heavily on the accuracy of the fitting template. The shape of the extracted suspected object image is different, so it is difficult to determine the fitting template. Modified moment method has good accuracy for centroid location of dim target and low computational complexity. Considering the complexity and accuracy of the algorithm, the modified moment method is used to locate the centroid of the suspected target. The calculation formula is as follows:

(20) $$\left\{ \begin{array}{c} centroid\_x=\sum_{x\in D}\sum_{y\in D}xI\left(x,y\right)/\sum_{x\in D}\sum_{y\in D}I\left(x,y\right)\\ centroid\_y=\sum_{x\in D}\sum_{y\in D}yI\left(x,y\right)/\sum_{x\in D}\sum_{y\in D}I\left(x,y\right) \end{array}\right.,$$

where (centroid_x,centroid_y) represents the centroid coordinate of the target, D is the target area, that is, the connected area of the suspected target, and I(x,y) represents the image used to calculate the centroid of the target. In order to avoid background interference, this paper calculates the centroid of the target using the image after background suppression. When the centroid of all suspected targets is extracted, all steps of GSO debris extractor are completed. It should be noted that we only extract the centroid information of the suspected target, but not the size, gray level, shape and other information, which is often an important basis for judging whether the target is a false alarm. This is because through the measured data testing, we found that the GSO debris extractor used in this paper can suppress background and stars very well. Among all the suspected targets extracted from a single frame image, the false targets are generally about 2 to 3, and in extreme cases, there will be no more than 10, so there is no need to eliminate false targets. In addition, because GSO debris are dim and small targets, if false alarm removal is performed, the GSO debris is likely to be deleted by mistake. Although we do not extract other information of suspected targets, the algorithm has good expansibility, and it is easy to extract other information in parallel while calculating the centroid.

## 3. GSO Debris Tracker

Track Association of GSO debris belongs to the field of multi-target tracking. There are mainly two kinds of multi-target tracking algorithms, one is based on RFS and the other is based on data association. Multi-target tracker based on RFS includes unlabeled RFS type and labeled RFS type. Unlabeled RFS type has the advantage of fast computation, but it does not have the ability to distinguish target tracks. Strictly speaking, it can be considered that the unlabeled label type is a target validator, and subsequent processing steps are needed to obtain target tracks. Labeled RFS type has the ability to distinguish target tracks, but it has a large amount of computation. In addition, all RFS trackers require that the target’s birth model be known prior, while in GSO debris tracking applications, the birth model is unknown. Multi-target tracking algorithm based on data association includes GNN, JPDA, MHT and their variants. In complex application scenarios, the tracking effect of MHT and JPDA is better than that of GNN, but the real-time performance of GNN is better than that of MHT and JPDA. Considering the simplicity of GSO debris motion, approximately stationary or uniformly low-speed linear motion, and the GSO debris extractor used in this paper can suppress background and stars very well, and the extraction results contain few false alarms. In this case, GNN multi-target tracker can complete GSO debris tracking task very well. Therefore, this paper uses GNN multi-target tracker to track GSO debris.

### 3.1. Adaptive Inter-Frame Interval Estimation

Before introducing the principle of GNN multi-target tracker, there is still a problem to be solved. Any tracker needs a relatively accurate time input, that is, the inter-frame interval is known. It is not necessary to consider this problem when the image sequence is of equal interval, but sometimes the exposure time is changed during GSO debris observation, which results in inconsistent inter-frame intervals. When the tracking error caused by time error is larger than the maximum error allowed by the tracker, it will cause the failure of target-track association at that time. The whole image sequence shows that the track is discontinuous, that is, the same target has multiple tracks. Therefore, it is necessary to extract the information related to the inter-frame interval from the image. Through analysis, the inter-frame interval can be represented by the translation vector obtained during image registration. Specifically, the inter-frame interval is the sum of charge transfer time and exposure time. The charge transfer time is much smaller than the exposure time, so the inter-frame interval can be approximated to the exposure time. When the telescope is in target tracking mode, the stars will form stripes on the CCD image. The stripes are caused by the rotation of the earth during exposure time, and the rotation speed of the earth is basically constant. Therefore, the exposure time can be estimated by the length of the stripes. The length of the stripes is positively correlated with the modulus of the translation vector, so the modulus of the translation vector can represent the exposure time. In summary, the modulus of the translation vector can indirectly represent the inter-frame interval.

In this paper, the translation vector modulus of 5 seconds exposure is taken as the reference modulus. The ratio of translation vector modulus obtained by adaptive fast registration algorithm to reference modulus is used as an adaptive estimation of inter-frame interval.

### 3.2. GNN Multi-Target Tracking Algorithm

This section introduces the principle of GNN multi-target tracker and gives a comparison with other trackers in GSO debris tracking applications. In the following description, $X_{o}=\left\{x_{o\_1},\cdots ,x_{o\_m}\right\}$ is used to represent the track table of the previous moment, where m is the number of targets. $\overset{⌢}{X}=\left\{{\overset{⌢}{x}}_{1},\cdots ,{\overset{⌢}{x}}_{m}\right\}$ represents the predicted track table at the current time, ${\overset{⌢}{P}}_{1},\cdots ,{\overset{⌢}{P}}_{m}$ represents the corresponding prediction error covariance matrix. Linear multiple-target motion model $X=FX_{o}+V=\overset{⌢}{X}+V$ is adopted, where $X=\left\{x_{1},\cdots ,x_{q}\right\}$ is the real track table at the current time, F is the state transition matrix, V is the process noise with zero mean and covariance matrix Q; then the transfer function can be expressed as $f\left(\overset{⌢}{x}|x_{o}\right)=N_{Q}\left(\overset{⌢}{x}-Fx_{o}\right)$. $Z=\left\{z_{1},\cdots ,z_{n}\right\}$ is used to represent the suspected target state table extracted at the current time, that is, the observation at the current time. The observation model is $Z=H\overset{⌢}{X}+W$, where H is the observation matrix, W is the observation noise with zero mean and covariance matrix R; then the likelihood function can be expressed as $f\left(z|\overset{⌢}{x}\right)=N_{R}\left(z-H\overset{⌢}{x}\right)$. Considering the Euclidean distance between observation z and prediction $\overset{⌢}{x}$, the uncertainty R of z and the uncertainty $\overset{⌢}{P}$ of $\overset{⌢}{x}$, referring to [42] (p. 95), the associated distance $d\left(z,\overset{⌢}{x}\right)$ between observation and track is defined by Bayesian method as follows:

(21) $$d\left(z,\overset{⌢}{x}\right)=\sqrt{{\left(z-H\overset{⌢}{x}\right)}^{T}{\left(H\overset{⌢}{P}H^{T}+R\right)}^{-1}\left(z-H\overset{⌢}{x}\right)},$$

GNN multi-target tracker is based on global associated distance. All possible associated distance between observation and prediction tracks are considered as follows:

(22) $$z_{\pi \left(1\right)}↔{\overset{⌢}{x}}_{1},\cdots ,z_{\pi \left(m\right)}↔{\overset{⌢}{x}}_{m},$$

where π:{1,⋯,m}→{1,⋯,n} is any possible one-to-one mapping. Therefore, the optimal association problem can be solved by solving π, which minimizes the following formula:

(23) $$d_{\pi }^{2}=\sum_{i=1}^{m}d{\left(z_{\pi \left(i\right)},{\overset{⌢}{x}}_{i}\right)}^{2}=\sum_{i=1}^{m}\left({\left(z_{\pi \left(i\right)}-H{\overset{⌢}{x}}_{i}\right)}^{T}{\left(H{\overset{⌢}{P}}_{i}H^{T}+R\right)}^{-1}\left(z_{\pi \left(i\right)}-H{\overset{⌢}{x}}_{i}\right)\right),$$

The mapping π obtained here is the optimal association between observation and prediction tracks determined by GNN multi-target tracking algorithm.

GNN multi-target tracking algorithm has low complexity and good tracking effect when the motion model is relatively simple. Let Dnum, Tnum and Fnum represent the number of observations, tracks and false alarms respectively, and assume that there is no target missed detection. Then the complexity of GNN tracking algorithm is $O\left(C_{Dnum}^{Tnum}Tnum!\right)=O\left(Dnum!/Fnum!\right)$. Since the motion form of GSO debris is relatively simple, GNN algorithm is adopted in this paper. In order to show the rationality of using GNN tracker in GSO debris tracking application, we record the processing time of GSO debris tracking using GNN tracker and other trackers in seconds, such as Table 1.

It should be noted that according to the processing results of the measured data, all the tracking algorithms used in the table can achieve better tracking results. There is no missing track in all tracking algorithms. There are three and one false tracks in the tracking results of PHD and CPHD algorithms, respectively. There are no false tracks in the tracking results of other algorithms. When the target track is established, the tracking algorithm based on RFS and MHT has a delay of 3–6 frames, and the GNN and JPDA algorithms have a delay of 3 frames. Seq_I–Seq_VII image sequences were tested. The numbers in parentheses represent the number of images in the corresponding image sequences. JointGLMB and GLMB are tracking algorithms based on labeled RFS. The contents in parentheses indicate the implementation method adopted. SMC represents sequential Monte Carlo method and GMS represents Gauss mixture method. CBMeMBer, CPHD and PHD are tracking algorithms based on unlabeled RFS, and their processing results need follow-up processing. In order to complete the test of the RFS tracker, we first analyze the data to obtain the birth model, and then use it as the input of the RFS tracker. This is obviously unreasonable in practical application. However, it should be noted that the unlabeled RFS tracker has a better processing speed advantage, and it is necessary to further study it, but this is beyond the content of this paper. MHT, JPDA and GNN are all tracking algorithms based on data association. When tracking applications are complex, MHT has the best performance, followed by JPDA and GNN. However, because the motion mode of target in GSO debris tracking application is relatively simple, and the target extractor used in this paper can suppress background and stars very well, there are a few false alarms in the extracted results. Therefore, there is no significant difference in the effectiveness of the three trackers. According to the above information, considering the tracking effect and real-time performance of the algorithm, it is reasonable to select GNN multi-target tracker for GSO debris tracking.

## 4. Results

### 4.1. Introduction of Measured Data

**Figure 9:**
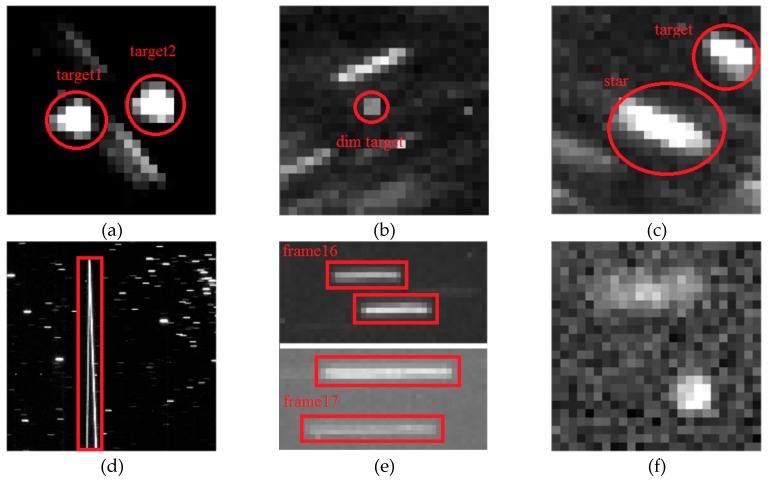
Typical cases of measured data: (**a**) close target; (**b**) dim target; (**c**) targets similar to stars; (**d**) high-brightness and fast-moving disturbing object; (**e**) exposure time change; (**f**) strong moonlight interference.

In order to test the algorithm comprehensively, we collected seven groups of image sequences by two telescopes, named Seq_I–Seq_VII. Seq_I–Seq_III was collected by telescope I, and Seq_IV–Seq_VII was collected by telescope II. The observation sites of telescope I and II are different, and their parameters are identical. The number of frames in each sequence is shown in Table 1. The exposure time of a single frame is usually between 5 and 10 seconds, so the frame rate is 0.1 to 0.2 frames per second. The images are all 16-bit gray-scale images of 2K×2K, and the single frame data is 8MB. We annotated ground-truth labels based on the public version of USSTRATCOM catalogue [44] and combined with manual screening. The characteristics of each image sequence are described as follows:

Seq_I, which contains two frames of irrelevant images, is located in the first frame and in the middle of the sequence, to test the adaptability of the registration algorithm in case of abnormality. It also contains two targets with very close distance. As shown in Figure 9a, the distance between two targets is less than 10 pixels, which can test the algorithm’s ability to distinguish close targets.

Seq_II, which contains more targets, is used to test the performance of the algorithm in the case of a large number of targets. Also, a new non-GEO debris enters the field of view, which can test the acquisition ability of the algorithm for the new-emerging target.

Seq_III is the typical data of Telescope I. It contains a very dim GEO debris, which can be used to test the limit detection ability of the algorithm. As shown in Figure 9b, the SNR of the target is very small. It also contains a non-GEO debris whose direction of motion is basically the same as that of stars, but whose velocity is smaller than that of stars. As shown in Figure 9c, the stripe direction of the target is basically the same as that of stars, but the stripe length is shorter than that of stars, which can be used to test the ability of the algorithm to detect targets with similar stellar motions.

Seq_IV image quality is poor. There are multiple frame dropouts and interference from fast moving bright object. As shown in Figure 9d, the interference target has a long bright smear, which can be used to test the performance of the algorithm in complex situations.

Seq_V, there is a change of field of view, testing the performance of the algorithm when the field of view changes. In addition, the exposure time changed once. As shown in Figure 9e, due to the change of exposure time, the length of the stellar stripe in frame 16 is shorter than that in frame 17, which can be used to test the adaptive ability of the algorithm when the exposure time changes.

Seq_VI, in which there is strong moonlight interference. As shown in Figure 9f, moonlight causes strong background noise, which can be used to test the effectiveness of background suppression algorithm.

Seq_VII is a typical data of telescope II, which can be used to test the performance of the algorithm under normal conditions.

### 4.2. Processing Results of Measured Data

**Figure 10:**
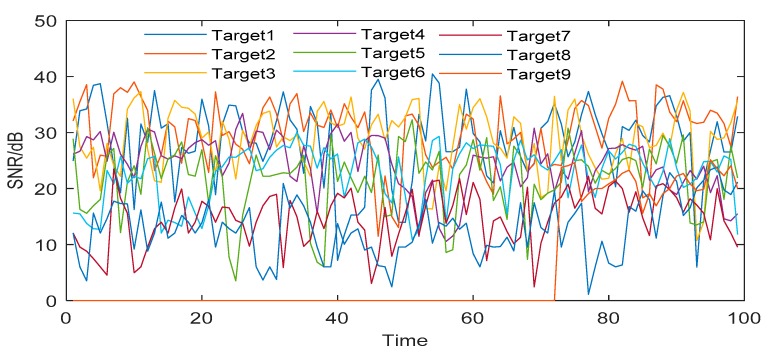
SNR of targets before image preprocessing.

**Figure 11:**
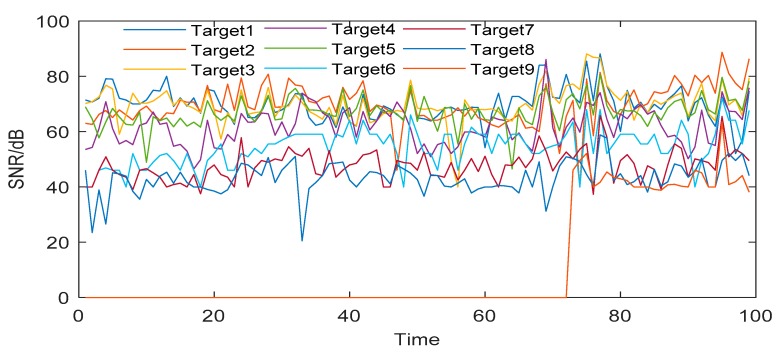
SNR of targets after image preprocessing.

All the tests in this paper are completed on Lenovo M410-PC with Intel i7-7700CPU@3.6GHz (8 CPUs), 8GB DDR4 RAM by using the software of MATLAB R2019(a). In order to show the detection ability for dim and small targets, we give the time-varying SNR of all targets detected in Seq_III, as shown in Figure 10, and the SNR of targets after image preprocessing, as shown in Figure 11.

**Figure 12:**
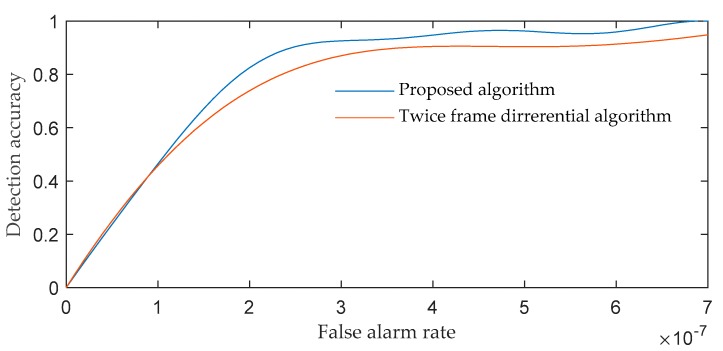
The receiver operating characteristic curves (ROC) of the proposed algorithm and the twice frame differential algorithm.

Figure 10 and Figure 11 show that the proposed image processing algorithm can effectively improve the SNR of the target, and then increase the detection probability of dim and small targets. Figure 12 shows the receiver operating characteristic curves (ROC) of the proposed algorithm and the twice frame differential algorithm [1]. The method of drawing the ROC curve is referred to [45].

Figure 12 shows that the proposed algorithm has better detection performance than the twice frame differential algorithm. Table 2 shows the number of GEO debris and non-GEO debris detected in seven groups of sequences, and the mean of SNR enhancement multiples of all targets in the sequence after preprocessing.

Table 2 shows that the algorithm can detect not only GEO debris, but also non-GEO debris, and at least can improve the SNR of detected targets by about 200 multiples. Table 3 gives the minimum SNR and missed detection accuracy of all detected GSO debris in seven sequences, in which SNR_min represents the minimum SNR of detected targets and Omi_R represents the missed detection accuracy. The definition of Omi_R in this paper is as follows:

(24) Omi_R=M_num/E_num,

where M_num denotes the number of frames that the target has not been detected, E_num denotes the total number of frames in which the target exists.

**Figure 13:**
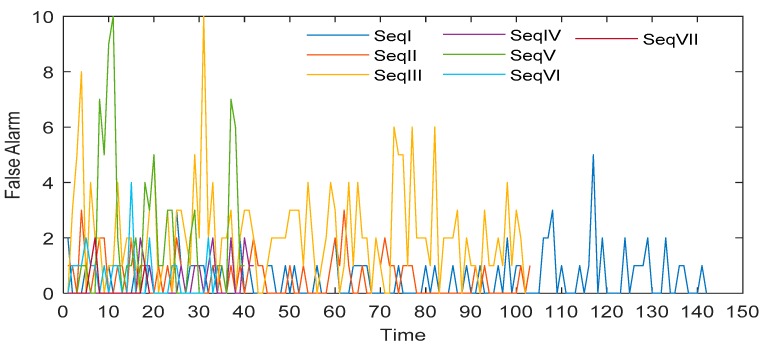
The number of false targets detected in each frame of seven image sequences.

Table 3 shows that the minimum SNR of the target detected by this algorithm is not more than 5.91 dB. At the same time, it should be noted that the missed detection accuracy of some targets is very high, so we relax the condition of judging the target track loss in the tracking algorithm. Only when the target is missed eight times in a row can it be judged that the track is lost. In addition, when the minimum SNR of different detectable targets is almost the same, the target miss detection accuracy is significantly different. This is due to the time-varying brightness of GSO objects. Because of the rotation and irregular shape of the object, the brightness of the object changes with time, and the change rate will be very large [21,22]. The intense change of brightness results in discontinuous detection results of targets, which leads to the above problems. For example, let the minimum SNR of the target detected by the algorithm be 5.9 dB. At k-time, the SNR of target A and target B are both 7 dB and detected. At k + 1, the target A changes bright, the SNR increases, the target B changes dark, and the SNR is less than 5.9 dB, which results in that A can be detected and B cannot be detected. That is to say, the minimum detectable SNR of A and B is 7 dB, but the missed detection accuracy is different. The number of false targets detected in each frame of seven image sequences is shown in Figure 13.

Figure 13 shows that the GSO debris extractor can extract no more than 10 false targets per frame, generally less than 4. This is because the extractor can suppress background and stars very well, and effectively enhance GSO debris. The processing time of each sequence is shown in Table 4.

**Figure 14:**
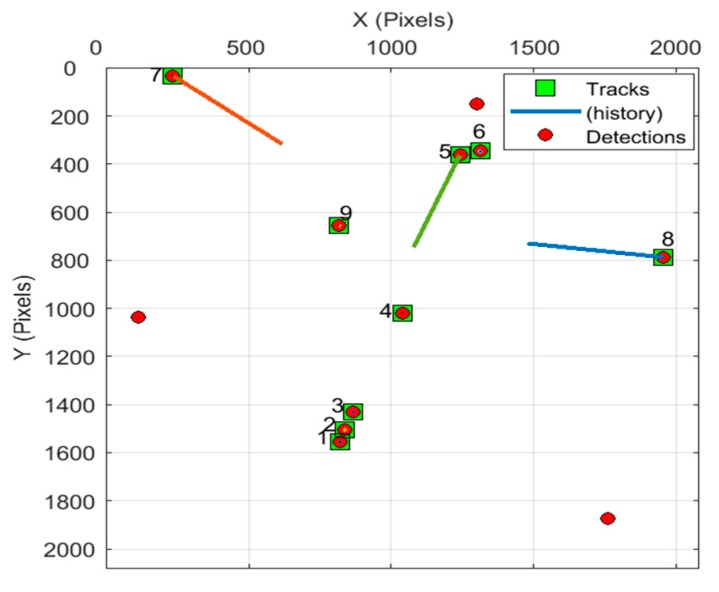
Seq_III detection result.

Table 4 shows that the single frame processing time of the proposed algorithm is not more than 2.112 s, while the exposure time of GSO debris observed with telescopes is generally more than 5 seconds, so the algorithm designed in this paper fully meets the real-time requirements of GSO debris detection tasks. In addition, there are differences in the processing time of single frame in Table 4, which is caused by the different stability of exposure time in image sequence. If the exposure time is stable, there is no need to use large-scale search frequently for registration, and the processing speed will be fast. However, if the exposure time changes frequently, it will lead to frequent use of large-scale search for registration, resulting in increased processing time. Finally, a representative Seq_III detection result image is given, as shown in Figure 14. Among them, color lines represent tracks, and different tracks are represented by different colors. The target with obvious track is non-GEO debris, otherwise it is GEO debris. The green rectangle represents the position of the target whose track has been established, and the red dot represents the position of the suspected target. Obviously, in the detection result of Seq_III, target 1, 2, 3, 4, 6, 9 is GEO debris and 5, 7, 8 is non-GEO debris.

## 5. Conclusions and Prospects

In this paper, an adaptive real-time detection algorithm for dim and small photoelectric GSO debris was proposed. It can effectively improve the detection ability of dim and small GSO debris. It has strong adaptability and does not require exposure time, telescope position and optical axis pointing information. Moreover, the real-time performance of the algorithm is strong, and it can fully meet the requirements of real-time processing ability in the application of GSO debris photoelectric detection. In addition, the algorithm has good parallelism. The algorithm consists of GSO debris extractor and GSO debris tracker. In the process of GSO debris extraction, an adaptive fast registration algorithm is proposed. The adaptability and real-time performance of the algorithm are effectively improved. In addition, in view of the shortcoming of the dilation difference algorithm, an enhanced dilation difference algorithm is proposed to improve the suppression effect for stars. For GSO debris tracker, an adaptive inter-frame interval technique is proposed to improve the performance. Using seven groups of measured data in different situations, the algorithm is tested comprehensively. The test results show that the proposed algorithm has the ability of self-adaptive detection of dim and small photoelectric GSO debris, and its processing speed fully meets the real-time requirement.

Some prospects for future work can be given. Firstly, because the algorithm has good parallel processing characteristics, it can be transplanted to embedded systems to further improve the processing speed. Secondly, in view of the theoretical advantages of RFS tracker, it is meaningful to find the method to solve the problem of unknown birth model in GSO debris tracking application with RFS tracker. Thirdly, considering that ISAL can image GSO debris, it can combine GSO optical detection system with ISAL imaging system to improve the recognition of GSO debris.

## Figures and Tables

**Table 1:** GEO debris tracking processing time-consuming by different trackers.

Time(s)	Seq_I (144)	Seq_II (105)	Seq_III (104)	Seq_IV (44)	Seq_V (42)	Seq_VI (36)	Seq_VII (21)
JointGLMB (SMC) [30]	189.523	130.008	108.813	29.544	19.893	28.419	13.075
GLMB(GMS) [43]	197.426	68.187	57.745	16.503	5.623	15.390	9.381
CBMeMBer (GMS) [29]	1.181	0.702	0.864	0.151	0.116	0.361	0.093
CPHD(GMS) [28]	1.005	0.795	0.745	0.186	0.167	0.264	0.101
PHD(GMS) [27]	0.51	0.484	0.516	0.084	0.093	0.172	0.066
MHT [33]	5.398	4.33	4.79	1.918	3.002	1.903	1.672
JPDA [32]	2.493	2.075	2.262	0.763	1.345	0.754	0.612
GNN [31] (pp. 203–205)	2.176	1.972	2.253	0.732	1.525	0.729	0.597

**Table 2:** The number of detected targets and the mean of SNR enhancement multiples.

Number	Seq_I	Seq_II	Seq_III	Seq_IV	Seq_V	Seq_VI	Seq_VII
GEO debris	6	7	6	4	4	5	3
Non-GEO debris	2	4	3	0	1	0	2
SNR Multiples	1313	1343	234	1390	595	539	780

**Table 3:** Minimum SNR and missed detection accuracy of detected targets.

Target	SNR_min (dB)	Omi_R (%)	Target	SNR_min (dB)	Omi_R (%)	Target	SNR_min (dB)	Omi_R (%)
Target1	9.85	0.00	Target17	27.12	0.70	Target33	17.43	4.35
Target2	13.4	0.00	Target18	10.26	1.41	Target34	19.53	4.35
Target3	14.14	0.00	Target19	13.21	1.41	Target35	22.61	4.35
Target4	14.23	0.00	Target20	15.45	1.41	Target36	25.71	4.35
Target5	14.78	0.00	Target21	17.06	1.41	Target37	10.71	4.90
Target6	16.32	0.00	Target22	22.96	1.41	Target38	16.7	6.52
Target7	16.99	0.00	Target23	7.37	1.96	Target39	13.33	9.38
Target8	19.28	0.00	Target24	14.35	2.63	Target40	17.13	12.50
Target9	20.5	0.00	Target25	8.24	2.82	Target41	**5.91**	16.67
Target10	23.06	0.00	Target26	6.98	2.88	Target42	10.91	17.65
Target11	24.22	0.00	Target27	7.68	2.88	Target43	15.81	19.05
Target12	25.31	0.00	Target28	18.27	2.88	Target44	6.024	30.39
Target13	29.89	0.00	Target29	7.19	2.94	Target45	24.56	40.66
Target14	33.08	0.00	Target30	10.55	2.94	Target46	17.69	53.33
Target15	33.5	0.00	Target31	9.97	3.13	Target47	12.49	**56.67**
Target16	24.46	0.70	Target32	11.48	3.92			

**Table 4:** The processing time of each sequence.

Time(s)	Seq_I	Seq_II	Seq_III	Seq_IV	Seq_V	Seq_VI	Seq_VII
Total	189.197	130.945	122.649	83.118	85.445	76.034	31.661
Single Frame	1.314	1.247	1.179	1.889	2.034	2.112	1.508
